# Pneumatosis Intestinalis and Aeroportia: A Case Report

**DOI:** 10.7759/cureus.45242

**Published:** 2023-09-14

**Authors:** Marcia Machado, Carlos Fernandes, Jorge Cotter

**Affiliations:** 1 Internal Medicine, Hospital da Senhora da Oliveira, Guimarães, PRT

**Keywords:** acute mesenteric ischemia, abdominal pain, intestinal ischemia, pneumatosis intestinalis, aeroportia

## Abstract

The presence of pneumatosis intestinalis (PI) and hepatic portal venous gas (HPVG) is associated with severe diseases. A 71-year-old man was admitted to the emergency department with complaints of severe and persistent nausea, vomiting, and diffuse abdominal pain that had been present for one week. An abdominal computed tomography (CT) showed aeroportia and PI, suggesting intestinal ischemia. Despite refusing an emergent exploratory laparotomy, the patient received medical treatment. However, due to the advanced stage of the condition, the medical treatment was ineffective, and the patient died a few hours later.

## Introduction

Pneumatosis intestinalis (PI) and aeroportia are rare radiological signs caused by gas infiltration in the intestinal wall and in the portal venous system, respectively. Separately, these two entities do not definitively indicate bowel ischemia. However, when they occur together, they are strong indicators of bowel ischemia [1] and are linked to a high mortality rate (85% of patients) [2]. The differential diagnosis of PI and aeroportia encompasses various diseases, including inflammatory bowel disease, gastric pathologies, diverticulitis, acute pancreatitis, invasive procedures, or intestinal ischemia [1,2]. Intestinal ischemia is a severe abdominal emergency that requires prompt diagnosis and treatment to prevent significant bowel infarction and improve prognosis [3]. When considering potential etiologies of intestinal ischemia, it is imperative to consider mesenteric ischemia due to its severity. Mesenteric ischemia can manifest by intestinal pneumatosis (6-43%) and aeroportia (3-36%) [4]. A contrast-enhanced abdominal computed tomography (CT) scan can identify the condition and underlying cause and detect any related complications [1].

We report a case of mesenteric ischemia accompanied by intestinal pneumatosis and portomesenteric venous gas. We aim to comprehensively review the current medical literature and clarify its clinical relevance.

## Case presentation

**Figure 1:**
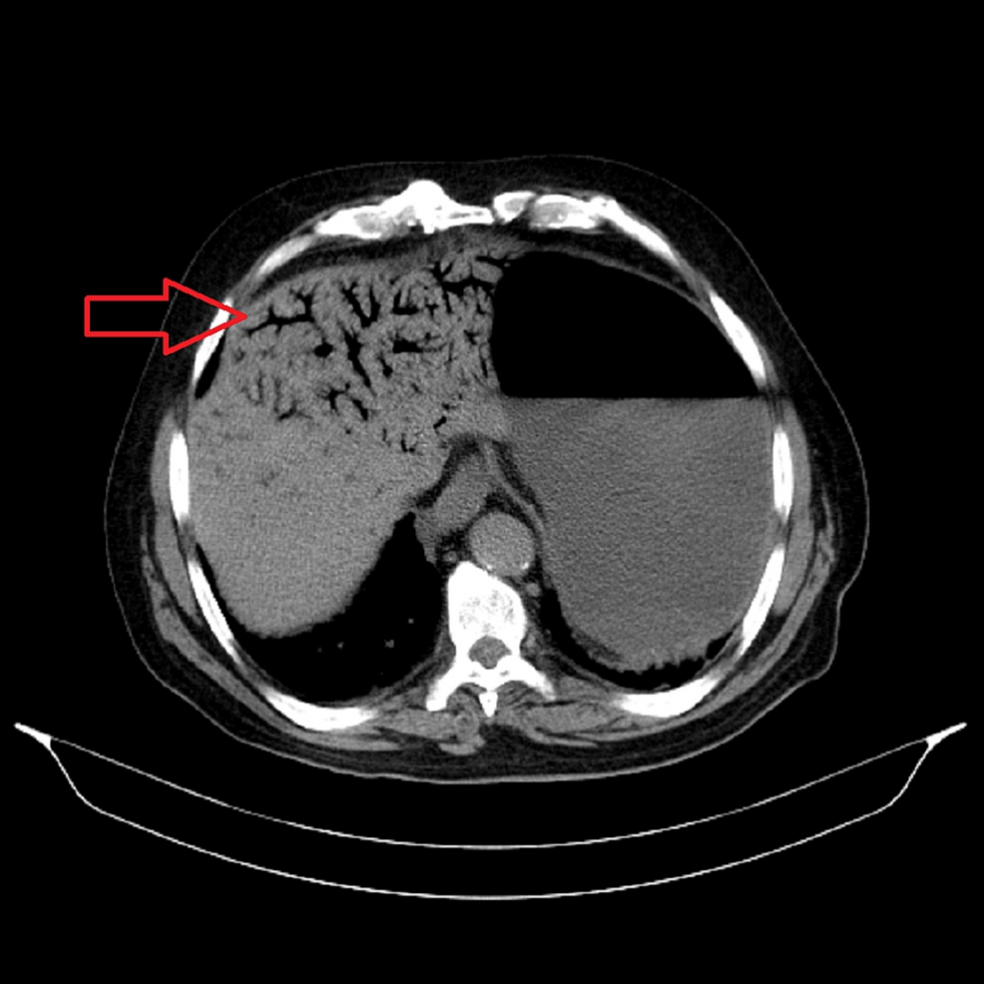
CT scan of the abdomen showing aeroportia (arrow)

**Figure 2:**
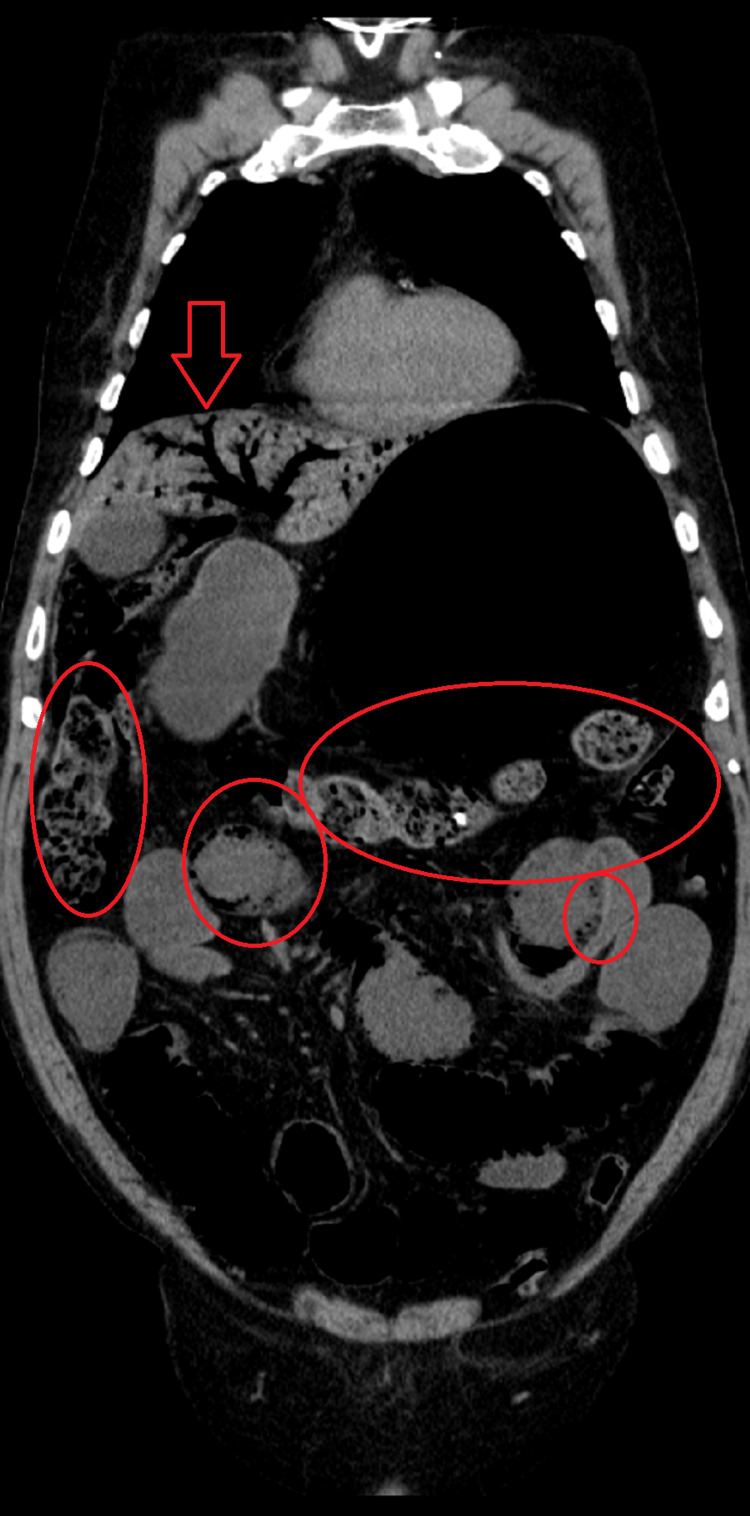
CT scan of the abdomen showing aeroportia (arrow) and intestinal pneumatosis (circle)

A 71-year-old man was admitted to the emergency department with complaints of severe and persistent nausea, vomiting, and diffuse abdominal pain that had been present for one week. He had a past medical history of hypertension, depression, and dementia and was taking the following medications: lisinopril/hydrochlorothiazide of 20/12.5 mg, escitalopram of 10 mg, quetiapine of 100 mg, olanzapine of 5 mg, and lorazepam of 2.5 mg. He had no surgical history. Physical examination revealed a patient alert but distressed. His pulse was 126 bpm, blood pressure was 84/53 mmHg, respiratory rate was 30 breaths per minute, and temperature was 38.5 ºC. During the examination, the patient's abdominal region exhibited distension and tenderness upon palpation, with absent bowel sounds. The patient had reduced peripheral pulses and increased capillary refill time. The patient was given broad-spectrum antibiotics (piperacillin/tazobactam) and 0.9% NaCl at 30 ml/kg. The blood test results revealed a hemoglobin level of 14.3 g/dL (normal range: 12-16 g/dL), creatinine level of 4.77 mg/dL (normal range: 0.7-1.3 mg/dL), urea level of 142 mg/dL (normal range: 15-39), and C-reactive protein level of 105 mg/L (<3 mg/L). Arterial blood gas revealed a pH of 7.32 (normal range: 7.350-7.450), HCO3 of 17 mEq/L (normal range: 22.0-31.0), anion gap of 27.0 mEq/L, and lactate level of 3 mmol/L (normal range: 0.5-2.2). Blood cultures were obtained and were negative. CT scan was performed, which revealed aeroportia as well as extensive intestinal pneumatosis of the jejunum and ileum, findings that were consistent with mesenteric ischemia (Figures 1-2).

The medical team suggested surgery, but the patient and their family declined. The patient was hospitalized with conservative treatment but died a few hours later.

## Discussion

When PI and aeroportia occur together, they are strong indicators of bowel ischemia [2]. This condition arises from the inadequate blood supply to the bowel that typically results from an embolism, thrombosis, or non-occlusive mesenteric ischemia. Symptoms can vary from mild abdominal discomfort to severe pain and diarrhea. The prognosis depends on etiology, but there is always a high mortality risk. A diagnosis can be made using an abdominal CT with contrast enhancement [5]. Although the physiopathology of intestinal pneumatosis is not yet fully understood, three theories attempt to explain it. The first is the mechanical hypothesis, which suggests that increased intraluminal pressure can damage the intestinal wall and cause gas infiltration. The second is the bacterial hypothesis, which proposes that certain bacteria in the intestinal wall produce gas. The third is the pulmonary hypothesis, which suggests that alveolar rupture due to chronic lung disease and increased thoracic pressure can cause gas infiltration into the bowel wall. There are two types of PI: primary form (which accounts for 15% of cases) and secondary form (which accounts for 85%). The primary entity consists of pneumatosis cystoid intestinalis, an idiopathic condition characterized by gas infiltration of the bowel and is usually benign. In contrast, secondary forms are associated with pathological patterns, such as acute intestinal ischemia, infections, recent abdominal traumas, iatrogenic gastrointestinal lesions, inflammatory bowel diseases, cancer, and immunosuppression. Thus, identifying the underlying cause is crucial for determining appropriate treatment and improving patient outcomes [1]. As with PI, gas in the portal vein is also not fully understood. Gas in the portal venous system may come from gas-forming organisms in the bowel or abscess, which spreads to the liver. It can also be caused by gas-forming portal venous system organisms entering the bloodstream [2].

As described, one of the causes may be acute mesenteric ischemia (AMI). AMI is a rare cause of acute abdomen, accounting for less than 1% of cases, and requires prompt intervention [4]. The diagnosis can be a challenge since symptoms can mimic less severe gastrointestinal diseases [6]. CT angiography (CTA) and magnetic resonance angiography (MRA) have greatly enhanced the accuracy of diagnoses [5]. The incidence of AMI increases with age, and it can be caused by mesenteric arterial embolism (50%), mesenteric arterial thrombosis (15-25%), or mesenteric venous thrombosis (5-15%). When there are signs of established intestinal infarction, the chance of survival is reduced dramatically. It is crucial to diagnose and intervene promptly to lower mortality rates that are above 50%. Conservative versus invasive treatment depends on the degree of ischemia and patient stability. Fluid resuscitation should be performed to improve visceral perfusion. Vasopressor therapy may be used, but with caution to avoid exacerbating ischemia. Due to the high risk of infection, it is recommended to administer broad-spectrum antibiotics. Despite this, surgery is the main treatment for acute occlusive mesenteric ischemia, but minimally invasive techniques such as endovascular interventions are also becoming more common. Intestinal viability is the most critical factor influencing outcome since a non-viable intestine, when unrecognized, results in multisystem organ dysfunction and death. The critical factors in ensuring survival include timely diagnosis, resuscitation, bowel viability assessment, vascular flow restoration, and non-viable bowel tissue removal [7].

The patient's survival was compromised due to a delay in seeking healthcare, as evidenced by this case. This case also emphasizes the need to educate the general public not to decline potential life-saving surgery in an emergency.

## Conclusions

Intestinal pneumatosis and aeroportia are rare but potentially severe medical conditions associated with various underlying conditions. Clinicians should be aware of the potential for intestinal ischemia in patients with abdominal symptoms and consider further diagnostic workup. Further research is needed to improve our understanding of this condition and to develop optimal diagnostic and treatment strategies.
